# Investigating the Classification of Living Kidney Donation Experiences on Reddit and Understanding the Sensitivity of ChatGPT to Prompt Engineering: Content Analysis

**DOI:** 10.2196/57319

**Published:** 2025-02-07

**Authors:** Joshua Nielsen, Xiaoyu Chen, LaShara Davis, Amy Waterman, Monica Gentili

**Affiliations:** 1 Department of Industrial Engineering JB Speed School of Engineering University of Louisville Louisville, KY United States; 2 Department of Industrial and Systems Engineering School of Engineering and Applied Sciences University at Buffalo Buffalo, NY United States; 3 Patient Engagement, Diversity, and Education Division Department of Surgery Houston Methodist Hospital Houston, TX United States

**Keywords:** prompt engineering, generative artificial intelligence, kidney donation, transplant, living donor

## Abstract

**Background:**

Living kidney donation (LKD), where individuals donate one kidney while alive, plays a critical role in increasing the number of kidneys available for those experiencing kidney failure. Previous studies show that many generous people are interested in becoming living donors; however, a huge gap exists between the number of patients on the waiting list and the number of living donors yearly.

**Objective:**

To bridge this gap, we aimed to investigate how to identify potential living donors from discussions on public social media forums so that educational interventions could later be directed to them.

**Methods:**

Using Reddit forums as an example, this study described the classification of Reddit content shared about LKD into three classes: (1) present (presently dealing with LKD personally), (2) past (dealt with LKD personally in the past), and (3) other (LKD general comments). An evaluation was conducted comparing a fine-tuned distilled version of the Bidirectional Encoder Representations from Transformers (BERT) model with inference using GPT-3.5 (ChatGPT). To systematically evaluate ChatGPT’s sensitivity to distinguishing between the 3 prompt categories, we used a comprehensive prompt engineering strategy encompassing a full factorial analysis in 48 runs. A novel prompt engineering approach, dialogue until classification consensus, was introduced to simulate a deliberation between 2 domain experts until a consensus on classification was achieved.

**Results:**

BERT and GPT-3.5 exhibited classification accuracies of approximately 75% and 78%, respectively. Recognizing the inherent ambiguity between classes, a post hoc analysis of incorrect predictions revealed sensible reasoning and acceptable errors in the predictive models. Considering these acceptable mismatched predictions, the accuracy improved to 89.3% for BERT and 90.7% for GPT-3.5.

**Conclusions:**

Large language models, such as GPT-3.5, are highly capable of detecting and categorizing LKD-targeted content on social media forums. They are sensitive to instructions, and the introduced dialogue until classification consensus method exhibited superior performance over stand-alone reasoning, highlighting the merit in advancing prompt engineering methodologies. The models can produce appropriate contextual reasoning, even when final conclusions differ from their human counterparts.

## Introduction

### Background

Kidney transplantation is the gold standard treatment for patients with end-stage renal disease [1] and can be much more cost-effective than dialysis [2]. Record numbers of transplants have taken place in recent years, but a shortage of donors continues to exist despite the recent increase [3]. Currently, the median wait time for a transplant is approximately 4 years in the United States, and approximately 5000 patients die every year while being on the transplant waiting list [4]. Living donor kidney transplantation (LDKT) generally provides better outcomes than deceased donor transplants but requires that a potential living donor be made aware that they can donate to a specific patient with end-stage renal disease and offer to do so. Racial or ethnic minorities and patients of lower socioeconomic status are less likely to pursue and have living donors donate on their behalf [5,6].

National attitudes about LDKT are generally positive, although many do not know what a living donor undergoes when donating a kidney [7-10]. Recommendations to increase the living donor pool include reaching out more broadly to locate generous individuals motivated by social good to engage more individuals in considering living donation [11]. In addition, research suggests that disseminating education and information about living donation to broader audiences, beyond transplant centers, might increase the numbers of potential donors and recipients pursuing living donation [12,13]. However, identifying individuals dealing with kidney disease and considering whether to pursue LDKT or donate kidneys in their own lives can be difficult, especially when they have not started medical evaluation at a transplant center.

Locating individuals through social media forums discussing living kidney donation (LKD), such as those on Reddit or Twitter (the work herein was done before the platform being rebranded as X), maybe a way to identify individuals who are actively deciding whether to pursue LDKT or LKD outside of transplant centers [14]. While there are many different types of questions and comments related to LKD shared on the web, some people share their personal experiences and even invite people to “ask me anything.” These findings motivated our main hypothesis that potential living donors can be identified from social media communities engaged in general discussions about LKD. In addition, understanding the personal experiences shared on these platforms can provide valuable insights into potential donors’ needs and decision-making, enabling education and media campaigns to be better tailored for them.

The large volume and high complexity of unstructured natural language require an effective and efficient method that can automate the identification of people sharing personal experiences with LKD. Fortunately, recent advances in natural language processing (NLP), particularly the transformer mechanism [15-19], enable the automatic understanding of personal experiences that were shared on the web social platforms. This study aimed to evaluate the transformer-based techniques to categorize these experiences on Reddit (Reddit, Inc). Specifically, we aimed to evaluate and compare (1) the one-shot classification model Bidirectional Encoder Representations from Transformers (BERT) [19], which required that we fine-tune the model using 1268 well-labeled samples, and (2) the zero-shot classification model ChatGPT (OpenAI), which required no fine-tuning for classification purposes. Comprehensive discussions on transformer-based models can be found in the study by Acheampong et al [20]. Much has been written about the capabilities and limitations of ChatGPT specifically [21]; however, we investigated the importance of prompt engineering when interfacing with it and other generative models applied to the field of organ donation for the first time.

### Overview of Prompt Engineering

Prompt engineering has been defined as “the means by which LLMs are programmed via prompts” [22]. Reynolds and McDonell [23] framed the objective of prompt engineering as a discipline that seeks to answer the question, “What prompt will result in the intended behavior and *only* the intended behavior?” Historically, the best practice has been to give a small number of examples of how the task is to be done, known as few-shot prompting. Ray [21] suggested that for large language models (LLMs), few-shot prompting is better thought of as “locating an already-learned task rather than meta-learning.” The implication is that the LLMs are large and robust enough that the models are inherently capable of completing NLP tasks, but their scale of capability may require using examples to “activate” the right parameters that will carry out the desired task in the prescribed manner.

However, this flexibility should also be understood as having dangers because LLMs can be “jailbroken.” Jailbreaking LLMs is the practice of using prompt engineering to work around the boundaries imposed by the developers, such as OpenAI [24]. The practice of “red-teaming” is used by developers to identify weaknesses in the desired boundaries and adjust the model so that it is more defensible against previous vulnerabilities [25,26]. What is simultaneously exciting and problematic about this is that many techniques used to jailbreak LLMs are the same as those used for their most helpful, intended uses, that is, many of the same methods that allow us to get the best performance from an LLM can be the same ones that are used to bypass the safeguards. Table 1 provides an overview of prompt engineering methods derived primarily from the study by White et al [22].

**Table 1:** Overview of prompt engineering methods proposed by White et al [22].

Method	Purpose	Example prompts for LKD^a^
Few-shot prompting	Provide examples that illustrate how the task is to be completed	“Here is an example of a risk analysis from a living kidney donation scenario: [EXAMPLE]. Now, please provide a risk analysis for the following scenario.”
Meta-language creation	Create a shorthand notation, abbreviated language, or set of standard rules	“For this conversation, ‘LKD’ refers to living kidney donation, ‘DT’ refers to donor testing and ‘RC’ refers to recipient compatibility. Using this shorthand, describe the typical process of LKD.”
Flipped interaction	The LLM^b^ will ask questions to obtain the information	“I’m working on an algorithm to match donors with recipients in living kidney donation. What information do you need from me to help design this algorithm?”
Persona	Assign a persona to the LLM, usually that of an expert	“Pretend you are a leading surgeon specializing in living kidney donation. Provide your expert opinion on the latest surgical techniques.”
Prompt refinement	Ensure that the LLM suggests better or more refined prompts	“I need to write code to analyze the success rates of different kidney matching algorithms. Could you suggest a more refined question or specific details you need to assist me?”
Alternative approaches	Ensure that the LLM offers alternative ways of accomplishing the task	“Describe three different methods for assessing donor-recipient compatibility in living kidney donation.”
Cognitive verifier	Subdivide a question into additional questions for a better answer	“To understand the ethical considerations in living kidney donation, what additional questions should I ask you to provide a comprehensive analysis?”
Fact checklist	Mitigate model hallucination by listing the facts	“After explaining the current trends in living kidney donation, list the facts or data sources you used in your response.”
Template	Ensure that the LLM’s output follows a precise template	“Please answer in the following format: ‘Living kidney donation is beneficial because [REASON 1], [REASON 2], and [REASON 3]’.”
Gameplay	Create a game around a given topic	“Let’s play a matching game. I will describe a recipient, and you suggest a suitable donor from the provided pool based on living kidney donation criteria.”
Reflection (chain of thought [25])	Explain the rationale behind the given answers	“Explain the process of donor selection in living kidney donation in a step-by-step manner, detailing the reasoning behind each step.”
Refusal breaker	Help users rephrase a question when they are refused an answer	“If you cannot provide personal patient data in living kidney donation, please guide me on how to rephrase my questions to obtain general information.”
Context manager	Enable users to specify or remove context	“When discussing living kidney donation statistics, please consider only data from the last five years in the European region.”
Recipe	Provide a sequence of steps given some partially provided ingredients	“I have patient medical records, compatibility testing results, and surgical schedules. Provide a sequence of steps to create an optimal living kidney donation matching algorithm.”

^a^LKD: living kidney donation.

^b^LLM: large language model.

Reflection and chain of thought reasoning, in particular, have garnered much attention due to their powerful results, creating what is already becoming a niche corner of research [27,28]. At the time of writing this paper and to the best of our knowledge, the 2 most recent and powerful of these improvements are the methods known as self-consistency [29] and the tree of thoughts [30]. The former uses majority voting from multiple replications, and the latter takes an ensemble approach to the chain of thought reasoning and allows LLMs to consider multiple different reasoning paths and to perform self-evaluation on choices. Other methods naturally exist beyond what is contained in this study because of the unbounded human imagination, which makes the domain of prompt engineering quite an exciting frontier. Interested readers may find the website [31] to be a useful resource, with new relevant articles being added to its repository regularly.

While prompt engineering in the context of LKD has not yet entered the literature, some work has emerged in the context of health care. Prompt engineering and generative artificial intelligence broadly are of particular interest in the medical domain as the generation of health information is still of unknown quality. A few researchers have emphasized the importance of medical professionals using LLMs skillfully and in a way that produces reliable information [32,33]. It has been shown that the reliability of GPT-4 (OpenAI) is inconsistent when answering medical questions, and the authors call for prompt engineering techniques to improve its performance [34]. Similarly, other authors have experimented with ChatGPT on calculation-based United States Medical Licensing Examination questions using 3 different prompting strategies, although they found that the prompt itself had only a small effect on answer accuracy [35]. Other research examined using prompt engineering in generating health messages [36] and even medical image segmentation [37].

### Social Media and LKD

Recent years have witnessed a burgeoning interest in studying dialogue on social media regarding important health care issues, such as vaccination [38] and LKD. Henderson [39] highlighted the use of platforms such as Facebook and Twitter to identify potential living donors while noting that formal research efforts are in their early stages. Analyzing social media content, including organ donation posts on the Chinese social media site Weibo, has unearthed key themes such as “organ donation behaviors,” “statistical descriptions of organ donation,” and “meaningfulness of donation” [40]. In one study, a notable 53% of potential living donors who self-referred for donor evaluation reported that they learned about a patient’s need for a donor on social media [41,42], while specialized tools such as the “DONOR” app have enabled expansion of social media marketing about living donation between potential donors and patients with kidney diseases [43]. Research efforts include measuring organ donation awareness through Twitter digital markers [44], surveying readiness of patients who are undergoing a transplant to use social media for education [45], and using Twitter for living donor profile classification [46].

Interventions to increase living donation have used mobile health technologies to manage donor follow-up [47], delivered targeted advertising to specific ethnic groups [48,49], and assessed organ donation awareness across the United States using Twitter data [50]. Best practices for promoting LKD through social media, such as delivering content to specific community demographics in targeted and interactive modes, have been proposed [51]; live transplant broadcasts on Twitter have occurred [52]; and the analysis of public Facebook pages of potential living donors [53] has enhanced insights into donor identification and donation interest. Recent studies highlighted the importance of tailored messaging over generic communication for better audience engagement [54,55].

These investigations underscore social media’s potential in augmenting donation awareness and facilitation, emphasizing the necessity for robust methods to discern and support individuals disseminating LKD-related content. A recent study by Garcia Valencia et al [56] has shown that ChatGPT can simplify medical information, making it easier to read and understand by many diverse groups. This can be a vital aid for promoting fairness in access to donation information from official sources. However, with the availability of *public* dialogue in forums also comes the need to thematically understand it. There is variation in both the content being shared and the user sharing it. The growing body of research demonstrates the potential of social media to impact awareness, intention to donate, and the facilitation of living kidney transplants. Therefore, it is necessary to have reliable methods whereby people who explicitly create and share content related to LKD can be automatically identified and understood for appropriate education and support. With this background, our research seeks to assess whether a classification system can be devised to discern individuals at varying stages of decision-making about becoming a living kidney donor. It also explores which of the contemporary NLP models are most apt for automating this classification, namely a fine-tuned distilled version of the BERT (DistilBERT) model (hereafter referred to as BERT for simplicity, unless greater specificity is merited) or ChatGPT. Furthermore, regarding ChatGPT, it examines how prompt engineering—namely, making adjustments to model instructions about the reasoning approach, examples, temperature, and class descriptions— influences its predictive efficacy for this application.

By answering these research questions, this study aimed to build a foundation for a sophisticated classification system in which it is possible to automatically categorize large amounts of social media communication about living donations using these tools. The study also aspires to gain a more in-depth insight into how individuals communicate and express themselves regarding LKD on various social media platforms. Using cutting-edge NLP technologies, our goal is to develop a streamlined, automated process for pinpointing curious, motivated potential donors who have not yet presented to the transplant center so that educational interventions could later be directed to them.

## Methods

### Data Labeling, Preparation, and Quality Assurance

We used a dataset of 2689 Reddit posts related to LKD from our previous work [14], which were published between January 2010 and April 2021. We also collected 603 Reddit posts from April 2021 to April 2023, for a combined total of 3292 posts from 2591 users. We scraped the posts with the open-source tool pushshift.io using keywords related to LKD, such as “kidney donor,” “kidney transplant,” “kidney donated,” “kidney donate,” “kidney years ago,” “kidney need,” “kidney stranger,” and “kidney willing donate.” Other search terms could have been included; however, as presented in Table 2, a considerable portion of collected data were not related to personal experiences, and we concluded that additional search terms would primarily expand the noise and add little value.

**Table 2:** Distribution and description of Reddit (Reddit, Inc) classes.

Merged class categories and class categories			Description		Example post
**Present (n=540, 26.9%)**					
	Present direct (n=363, 21.5%)	The user has *current firsthand experience* with something personally related to kidney disease, kidney failure, living kidney donation, or transplantation (eg, the user with kidney disease or kidney failure, is on dialysis, is seeking a kidney, is exploring donation, or is undergoing evaluation for donation or transplantation).		“A friend of mine is in need of a kidney. My first instinct is to offer one of mine. I have Googled and read LOTS of info. What would you do? Have you donated a kidney? What am I missing?”	
	Present indirect (n=177, 5.4%)	The user has *current secondhand experience* related to living kidney transplantation (eg, they *know someone* who is currently experiencing kidney failure, on dialysis, seeking a kidney, or preparing to donate a kidney).		“I need help finding a kidney for my dad.”	
**Past (n=222, 6.8%)**					
	Past direct (n=168, 5.1%)	The user has *past firsthand experience* related to living kidney transplantation (eg, kidney failure, dialysis, kidney recipient or donor).		“Eight years ago today, I donated a kidney to a friend. Ask me anything.”	
	Past indirect (n=58, 1.8%)	The user has *past secondhand experience* related to living kidney transplantation (eg, they *know someone* who experienced kidney failure, was on dialysis, received a kidney, donated a kidney, underwent evaluation for donation, or participated in the donation process (perhaps in a supporting role).		“Picture of my dad and the woman who donated a kidney to save his life.”	
**Other (n=2530, 76.8%)**					
	General commentary or hypothetical (n=159, 4.8%)	The user is giving a *general opinion* on the topic, asking a *hypothetical question*, or contributing to discussion about an *imagined scenario*.		“If you donate a kidney, then later your only one starts to fail, would you be put on a higher priority?”	
	News or noise (n=2371, 72%)	The user is either sharing a *news article or headline* related to kidney donation that may be pertinent but *not personal*, or it is *simply irrelevant*.		“A man donated his kidney to his wife of 51 years after finding out he’s her perfect match.”	

We selected Reddit as our data source because it provided the greatest portion of comments that were related to personal experiences rather than discussions of policies and sharing news stories. Reddit was the only place where we found posts from actual living donors inviting people to an “ask me anything” session, sparking highly personal discussions [14].

Under the guidance of LKD domain experts, after reviewing 100 example posts, we created 2 class sets, one with 6 classes (class categories) and the other with 3 classes (merged categories), to automate the process of identifying firsthand experiences with living donation (Table 2). These classes were iteratively defined and improved through multiple discussions with a team of 6 people who performed the manual annotation. Certain posts had sufficient ambiguity to make an explicit ruling impossible. For example, it was not always clear what constituted the boundary between a past and present experience (eg, how much time should have passed since the transplant?) or whether the general transplant mentioned in a post came from a living or deceased donor. Furthermore, long and verbose posts with brief mentions of personal experiences with donation posed a challenge because the brief (although important) mentions of LKD were easy to miss. Individual annotators were found to exhibit varying classification tendencies or use their own “rules of thumb” to expedite the often tedious process.

The granularity between these 6 fine-grained classes proved quite difficult for the models to correctly capture during initial experiments (resulting in accuracies <50%), so the posts were consolidated into the 3 coarse-grained categories: present (n=540, 42.59% of posts), past (n=222, 17.51% of posts), and other (n=506, 39.91% of posts randomly sampled from news or noise and general commentary or hypothetical categories) for 1268 samples that were used for training the BERT model. A randomly selected subset of 100 from each of the 3 classes was used for prompting with ChatGPT. The decision was made to aggregate general commentary and hypothetical posts with news or noise to ensure a more precise focus on personal experiences.

Acknowledging the potential data quality risks [57], we meticulously evaluated incorrect predictions from both BERT and ChatGPT after the analysis. The incorrectly predicted samples were tagged as either acceptable errors (reasonable, if not perfectly aligned predictions), unacceptable errors (flawed or evidently incorrect reasoning), more accurate than the original human label, or instances where both human and model erred. We later reported these using the notation of *LLM≃human*, *LLM<human*, *LLM>human*, and *both error*, respectively, for both models.

### Ethical Considerations

This study was granted an exemption from The University of Louisville Institutional Review Board (review number 22.0458). While there could be ethical concerns about consent and storage of health-related data, every Reddit user is entirely anonymous, ensuring that nothing we find can be directly traced to an individual. In addition, the comments and posts themselves are all very public; some websites may have minimal requirements, such as logging in or being a member of a “closed” group before the content can be observed; however, this is not the case for any of the data we collected. For data sources where such anonymity is not guaranteed, it is imperative to ensure that users consent to the study of their created content and that any identifying information be removed or obscured.

### Modeling

We compared 2 transformer-based models for our classification task: a fine-tuned BERT model and a prompt-engineered ChatGPT model. We used the 3.5 Turbo version of ChatGPT via the OpenAI application programming interface and conducted a full factorial analysis of various prompt components to identify the best features. The DistilBERT model was fine-tuned from a pretrained Hugging Face (Hugging Face, Inc) model. Furthermore, we noted that many new models have emerged, both proprietary and open source, after our experiments were completed. Post hoc experiments indicate that our findings are consistent with newer models.

### BERT Analysis

The DistilBert tokenizer from Hugging Face was used to tokenize the text data from Reddit, and both input IDs and attention masks were generated to structure the text inputs for the model. A custom model was designed around DistilBERT. The architecture included the pretrained DistilBERT model, followed by 3 fully connected layers with 768, 256, and 128 units, respectively. These were followed by an output layer with 3 units corresponding to the number of classes. Batch normalization and rectified linear unit activation functions were applied, and dropout was set at 10%.

The focal loss was used as the loss function, which is designed to address the class imbalance by downweighting the loss assigned to well-classified examples [58]. It was parameterized with an α factor for controlling the weight and a γ factor for focusing on hard examples. The model was trained using the AdamW optimizer [59], with the learning rate and weight decay optimized by the open-source Optuna hyperparameter tuning library. The dataset was split into training and validation sets using stratified 5-fold cross-validation, with class weights computed to manage class imbalance, and the model was trained for 3 epochs, following the recommended fine-tuning procedures [19]. The metrics used for validation are defined subsequently.

Accuracy is the ratio of correctly predicted instances to the total instances.



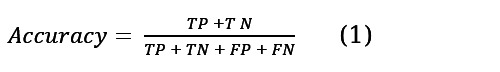



Precision is the ratio of correctly predicted positive observations to the total predicted positives.



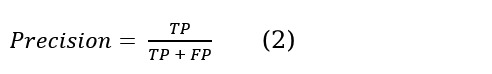



Recall (sensitivity) is the ratio of correctly predicted positive observations to all observations in actual class.



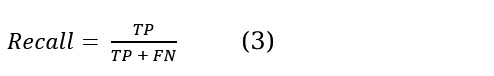



*F*_1_-score is the harmonic mean of precision and recall.



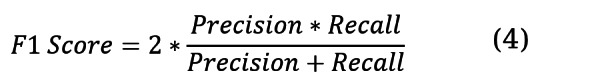



In equations 1 to 4, *TP*, *TN*, *FP*, and *FN* are the numbers of true positive, true negative, false positive, and false negative values, respectively.

The Optuna library was used to perform hyperparameter optimization, which uses a Bayesian optimization method known as the Tree-structured Parzen Estimator [60]. A search space was defined for the learning rate (ranging from 0.00003 to 0.0003) and weight decay (ranging from 0.0001-0.001). A total of 100 trials were conducted to find the best set of hyperparameters based on the *F*_1_-score.

### Dialogue Until Classification Consensus

We introduced a text classification tool for LLMs termed “dialogue until classification consensus” (DUCC). Given the absence of a formal taxonomy for prompt engineering methods, we aligned DUCC’s presentation with the pattern widely adopted in software development, which includes a name and classification, intent and context, motivation, structure and key ideas, example implementation, and consequences (Textbox 1). White et al [22] constructed the following categories of prompting patterns: input semantics, output customization, error identification, prompt improvement, interaction, and context control.

**Prompting patterns for “dialogue until classification consensus” (DUCC).**

**Name and classification**

DUCC primarily falls under output customization, although it shares elements from other pattern categories, notably error identification and interaction.

**Intent and context**

DUCC assigns a persona of at least 2 domain experts to the large language model, instructing them to discuss a text sample until a consensus on its classification or answer selection is reached from a set of options. This setup aims to automate explicit reasoning and reflection through a simulated dialogue, expecting to resemble the effects of distribution-oriented methods, such as self-consistency, without requiring multiple sample replications.

**Motivation**

Complex classification tasks, especially within niche domains, such as personal living kidney donation experiences, often present labeling challenges. DUCC simulates expert discussions for decision-making while aiming to standardize output formats for classification tasks.

**Structure and key ideas**

Experts 1 and 2, specialized in [DOMAIN], are to discuss the text sample until an agreed classification or answer is reached.

The final label should be clear with no disagreements, formatted as: “classification: Label.”

Additional identities or traits can be attributed to the experts to infuse specific perspectives into the discussion. We have observed that unless a singular label selection is emphasized, the model might assign multiple labels in challenging scenarios.

**Example implementation**

“Expert 1 and Expert 2, you are both experts in living kidney donation, and you’ve been tasked with analyzing and classifying a Reddit post that should be related to living kidney donation. You should discuss the post until you come to an agreement for a single classification. If the post is not related to living kidney donation, it needs to be labeled ‘Other’. The classifications are defined as follows:

- Present: The user is describing a current or ongoing personal experience with living kidney donation
- Past: The user is describing a past personal experience with living kidney donation.
- Other: The user isn’t discussing a personal experience with living kidney donation or isn’t discussing living kidney donation at all.

Discuss until you reach a consensus, showing your reasoning. The final label should be clear, and there should be no disagreement. Output your agreed label in this format: {‘classification’: ‘your agreed label’}.

Here’s an example of how this should be done:

- Post: ‘Are you a kidney donor? How was the recovery process and how are you doing now?’
- Expert 1: ‘I think the appropriate label is Present, because the user is asking questions and seems to want information to help them with a current decision about living kidney donation.’
- Expert 2: ‘I think the appropriate label is Past because the user wants to know about past personal experiences from others.’
- Expert 1: ‘I see your point about bringing up the past, but since we are interested in assigning a label to the user who wrote the post, we should keep our focus on the author’s perspective. If we knew what the replies were, we could label those users as Past, but we are only looking at this user for now.’
- Expert 2: ‘You’re correct, we should be focused on this user rather than possible answers from others. Even though there are elements of both, we have to pick one and only one label, so let’s go with Present.’
- Final Label: ‘‘classification’: ‘Present.’”

**Consequences**

DUCC prompts large language models to reason through multiple perspectives, ensuring a singular, consistently formatted label, simplifying extraction. The example implementation is crucial as it demonstrates the desired dialogue structure, aiding the model in handling nuanced classifications. However, DUCC may exhibit biases when numerous classes are present, potentially leaning toward the exemplified label. To mitigate token use, especially in lengthy examples, using DUCC when defining the system instead of individual prompts is advisable. For instance, in the OpenAI application programming interface, modifying the “content” section of the “system” role with the entire provided example instead of the default content can better define the system’s nature.

### Sensitivity Analysis of Prompting

#### Overview

For our experimentation using ChatGPT to categorize personal experiences, we conducted a study applying a full factorial design with 4 factors (summarized subsequently), which resulted in 48 experimental runs. We must first acknowledge that the nature of prompting is such that there were an infinite number of ways we could write the prompt and parameters that could be chosen. It is well known that examples that illustrate the solutions can influence performance (known as “few-shot” prompting) [61], so we examined the number of examples and the type of examples that might produce bias as well as the parameters provided subsequently.

#### Use of the DUCC Method (2 Settings)

In addition to the DUCC method described earlier, the alternative was to prompt a single expert to make a classification decision, with the instruction to “Examine the evidence for each class option step by step. The final label should be clear.” In this case, the model attempts to identify any evidence that suggests the sample should be assigned to each class and weighs the evidence to draw a conclusion.

#### Number of Examples Used (4 Settings)

We selected either 1 example or 3 examples. For 3 examples, 1 example was used for each class (present, past, and other). For the single example setting, we performed an experiment with each class once to evaluate whether it produced a bias in the predicted class.

#### Definition of “Past” (2 Settings)

Observing a tendency for underprediction in the “Past” label, we considered 2 definitions for the class. The first was a short and concise definition: “The user is describing a past personal experience with living kidney donation.” The second was a longer, more descriptive definition: “The user is referring to a past personal experience with LKD. This may be presented in the context of a present tense story, but if the event of LKD was lived previously, the post should be labeled past.”

#### Temperature Settings (3 Settings)

Experimentation spanned temperature values of 0, 0.15, and 0.3, investigating the tradeoff between output variability and consistency. The settings were guided by OpenAI documentation, emphasizing lower values for consistency and higher values for diversifying outputs [62].

Given the cost implications of OpenAI application programming interface calls, an initial assessment was carried out to determine the necessity for replicating each setting. We performed 30 replications of a fixed parameter setting and found no substantial effect within replications for any metric. Thus, the experimentation proceeded with a singular sample for each parameter setting.

## Results

### Overview

In this section, we present the results of the BERT model first and then the results of ChatGPT. We present the performance metrics, confusion matrices, and assessment of incorrect predictions. For ChatGPT, we also present the results of an ANOVA on the various factors used in the experimentation.

### BERT Results

**Figure 1:**
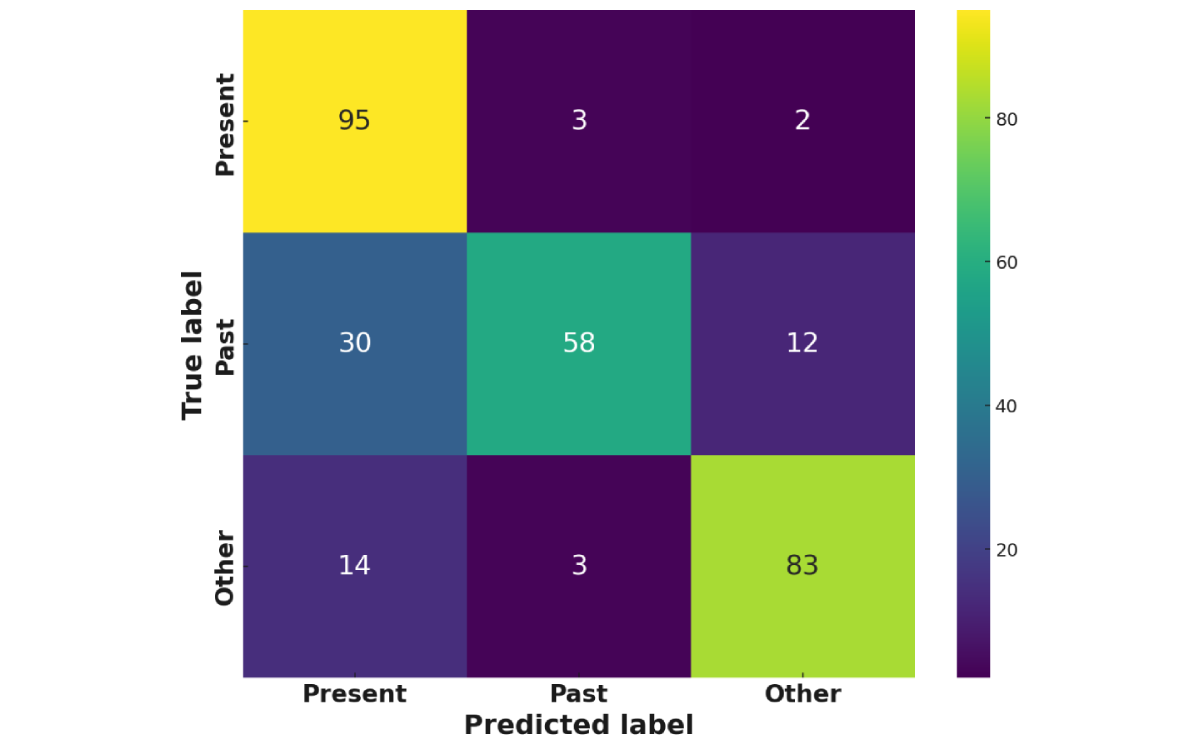
Confusion matrix for the best Bidirectional Encoder Representations from Transformers model.

In >100 trials, the best BERT model performed with an accuracy of 75.1% and an *F*_1_-score of 78.2% on the validation data during training. The best parameters were a learning rate of 0.000131687 and a weight decay of 0.000791. The confusion matrix for the predictions on the test data is presented in Figure 1, showing reasonably good performance but with a tendency to erroneously predict the Other label on both past and present labels.

The classification report provided in Table 3 shows that the BERT model significantly underpredicts past labels, partly due to the smaller sample size, and also because of the ambiguity that can arise when a reference to a past experience is nested within an ongoing story.

**Table 3:** Classification report.

	Precision	Recall	*F*_1_-score	Support
Present	0.88	0.82	0.85	101
Past	0.66	0.52	0.58	44
Other	0.75	0.86	0.80	108
Weighted average	0.79	0.79	0.78	253

### ChatGPT Results

The best ChatGPT prompt produced an accuracy and *F*_1_-score of 78.67% and 78.17%, respectively (surprisingly, this *F*_1_-score is identical to that of BERT). This was achieved using the DUCC method, a single example of a present class post, a temperature of 0, and the shorter definition of the past class (refer to the Dialogue Until Classification Consensus section). Full experimentation results are provided in the Multimedia Appendix 1. The next 3 columns show the percentage of predictions for that class, and the remaining 3 columns show the evaluation metrics.

**Figure 2:**
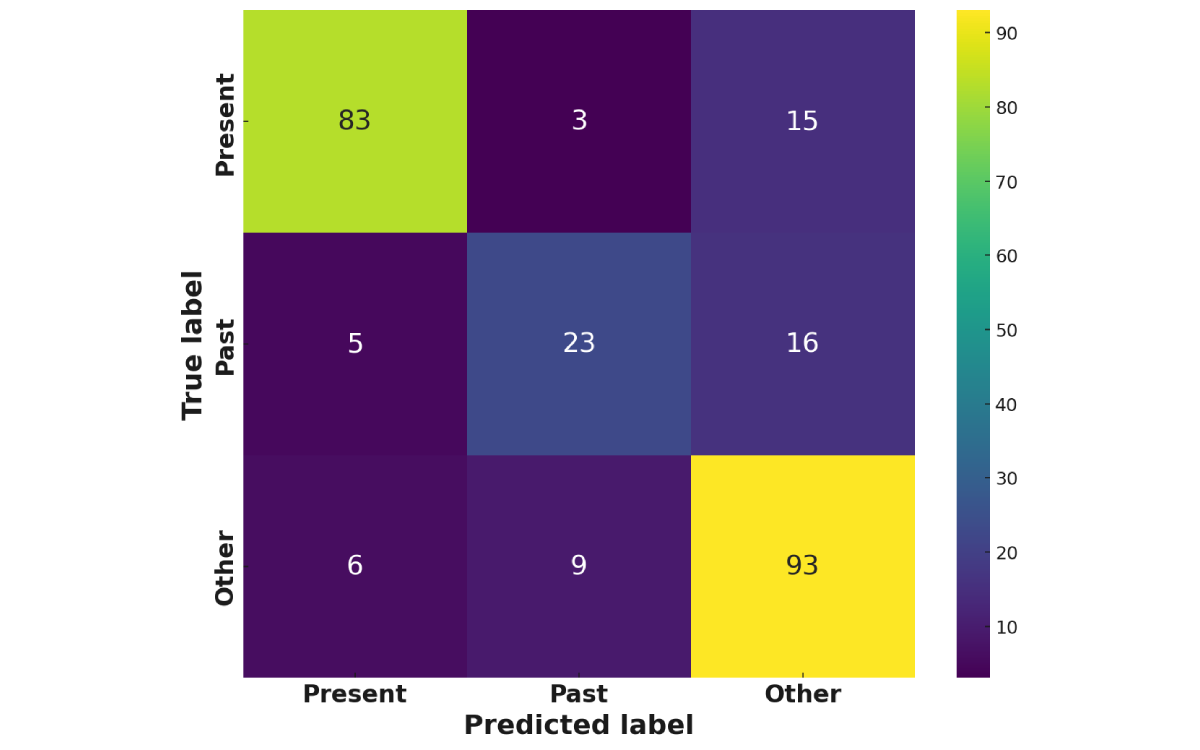
Confusion matrix for the best ChatGPT prompt.

The confusion matrix for ChatGPT performance is presented in Figure 2, which shows again that past class samples were underpredicted and that both other and past class samples were overpredicted to be present class, suggesting a bias toward present classifications.

The results of the ANOVA are presented in Table 4, which shows that the number and type of examples used is the most significant factor, followed by the method. We observe that the examples and method factors were the only statistically significant factors.

Given that there were 3 df within the examples setting, we sought to better understand the difference between the example settings using a Tukey test, with results provided in Table 5. We observed that when our example belonged to the “past” class the model performed better than when the example came from the “other” class. But using an example from the “past” class resulted in poorer performance compared to using 3 examples (one from each class) and using an example from the “present” class. Interestingly, the “past” sample was underpredicted in every setting except when using 3 examples and the evidence method. Interestingly, samples belonging to the “past” class were underpredicted in every setting except when using 3 examples and the evidence method. Although this setting (3 examples; evidence method) does not demonstrate the same underprediction bias as other settings, it does not give better accuracy overall.

**Table 4:** ANOVA results.

Factor	Sum of squares	*F* test (*df*)	*P* value
Category (examples)	0.068615	27.659884 (3, 40)	<.001
Category (method)	0.006466	7.819650 (1, 40)	.008
Category (temp)	0.000024	0.014557 (2, 40)	.99
Category (past)	0.000032	0.039292 (1, 40)	.84
Residual	0.033076	—^a^	—

^a^Not applicable.

**Table 5:** Multiple comparisons of means using the Tukey honestly significant difference test. The family-wise error rate is 0.05.

Group 1	Group 2	Mean difference	*P* value	Lower limit	Upper limit	Reject
1, other	1, past	–0.0875	<.001	–0.1202	–0.0548	True
1, other	1, present	0.0078	.92	–0.0249	0.0405	False
1, other	3	–0.0017	.99	–0.0344	0.031	False
1, past	1, present	0.0953	<.001	0.0626	0.128	True
1, past	3	0.0858	<.001	0.0531	0.1185	True
1, present	3	–0.0094	.87	–0.0421	0.0233	False

## Discussion

### Principal Findings

Our experimentation has found that BERT and ChatGPT perform comparably for the classification of different living kidney donor experiences. Because BERT is completely dependent on the available training data, ChatGPT can be used with a somewhat higher degree of precision via prompt engineering, as shown by our use of the novel DUCC method. Our full factorial experimentation identified the best settings to use for our engineered prompt. In this section, we will discuss the predictions that were made incorrectly and consider future work and ethical considerations.

### Examination of Incorrect Predictions

As noted in the Data Labeling, Preparation, and Quality Assurance section, there is an inherent risk of data quality that arises from the dataset in question. Unlike standardized benchmarks, which often have explicit “ground truth” labels, our task is fraught with nuance. Despite our extensive efforts to ensure data quality, the given label is not always clear. As such, we have provided a more detailed examination of the instances where the models made predictions that diverged from the given labels.

BERT and GPT-3.5 produced 21.3% (54/253) and 21.3% (64/300) incorrect predictions, respectively. It should be recalled that the difference in the denominator values is because BERT requires a split test set, whereas, with GPT-3.5, we can use a larger inference-only set. We assessed the quality of these incorrect predictions not only to see how “close” they were to the mark but also to determine whether any human errors had been made in labeling the incorrect predictions. As provided in Table 6 for BERT, we observe that 27 prompts were incorrectly labeled either because of an acceptable error where a clear prediction is difficult to make (perhaps due to the ambiguity of what constitutes the difference between the past and present samples) or where BERT made a better prediction than the original human label. Treating these 27 predictions as being acceptable or correct brings the total number of correct predictions from 199 (78.7%) of 253 to 226 (89.3%) of 253, which elevates the predictive accuracy considerably to 89.3%. In these tables, examples are written “as they are” from the original posts, including typos and terminology that may be unique to Reddit.

**Table 6:** Analysis of incorrect predictions from Bidirectional Encoder Representations from Transformers (BERT; n=54).

Error type	Incorrect predictions, n (%)	Example post	Reason
Unacceptable error (BERT*<*human)	22 (41)	“Required testing to be a living Kidney donor where I live - these are the tests I took before becoming a living kidney donor almost 2 yrs ago everything has gone great for me and the recipient happy to answer any questions.”	BERT predicted the “other” label, but the user clearly states that he or she was a previous living donor.
Acceptable error (BERT≃human)	12 (22)	“Hey Mum, it’s been a year since what was supposed to be a life changing kidney transplant that took a turn for the worst. I love you so much and think about you every day xxx”	BERT predicted the “other” label, which could be appropriate if it was a deceased donor transplant. We predicted the “past” label.
Human error (BERT*>*human)	15 (27)	“Me 26F with my Dad 58 he needs a kidney and I feel pressured to donate one. [removed]”	We predicted the “other” label because of the (removed) tag at the end of the post, which commonly appears in unusable posts. BERT predicted the “present” label, which is the more appropriate label.
Both erred	5 (9)	“I used to like her but I found out that she did not even acknowledge her kidney donor... Just referring to her as a person I know it seems pretty ungrateful [removed]”	This is someone’s opinion about a celebrity who famously received a kidney transplant from her friend. It is not a personal experience at all, but the human label was “present,” and the BERT label was “past.”

From our analysis of the incorrect predictions on GPT-3.5 (Table 7), we observed that 26 (40%) of the 64 errors were acceptable.

As mentioned earlier, we had previously observed that many “past” posts were labeled as “present” because many of the posts were in a present tense context. The best setting used the shorter definition of past, which does not teach the model to treat past experiences nested in present accounts as the past class, so this is to be expected. Anytime both the human and predicted labels were wrong, the post was almost always ambiguous regarding whether it was about living or deceased donation. The experiences being described could have been a living donation, but there is not enough information to determine that for certain.

Regarding BERT, we may allow ourselves to consider the 26 acceptable errors and 10 human errors as being correctly predicted, changing the total number of correct predictions from 236 (78.7%) of 300 to 272 (90.7%) of 300 for an “actual” predictive accuracy of 90.7%. While still imperfect, this shows considerable reliability when using these methods on nuanced language tasks.

The implications of this examination are threefold: (1) sometimes human annotations go wrong, even with clear instructions; (2) these powerful models are capable of correctly catching things that humans miss (due to decision fatigue or similar cognitive difficulties); and (3) the models can be largely trusted to give sensible reasoning, even if the final conclusions differ from that of a human counterpart.

**Table 7:** Analysis of incorrect predictions from ChatGPT (n=64).

Error type	Incorrect predictions, n (%)	Example post	Reason
Unacceptable error (ChatGPT*<*human)	21 (33)	“relationships My (36F) estranged sister (43F) donated a kidney to me. I just heard that she died (for a different reason). I’m very confused. [removed]”	The simulated experts reasoned that the focus of the post was on grief rather than LKD^a^ and labeled it as “other.” The human label was given as “past” because the user mentions a sister who donated her kidney some time ago.
Acceptable error (ChatGPT human)	26 (41)	“Successfully donated a kidney to my sister whos been fighting Lupus.”	This could be easily interpreted as either a “present” (ChatGPT) or a “past” (human) label, given that there is no explicit reference to time. It could go either way, but it is still clearly related to a personal experience with LKD.
Human error (ChatGPT *>* human)	10 (16)	“I (30F) had heart and kidney transplant. Ask Me Anything (AMA).”	The simulated experts concluded that this should be labeled “other” when the human label had been given as “past.” ChatGPT made a more correct conclusion because this may have been from a deceased donor rather than a living donor. We would need more information to be certain, so it should be an “other” label.
Both erred	7 (11)	“I am A double kidney transplant recipient! AMA! I am a 28 year old white male, I’ve had two renal transplants over the course of my lifetime. I’ve been on dialysis. I’ve been in and out of hospital my entire life. I think it’s interesting, but there’s only one way to find out! Ask Me Anything.”	The human-given label for this was “past” because of the previous transplant experiences, and the reasoning provided by ChatGPT concluded that the label should be “present” because the user mentions dialysis and being in and out of the hospital. Both were incorrect because there is not enough evidence that either of the transplants was from living donors, and thus, it should be labeled “other.”

^a^LKD: living kidney donation.

### Limitations and Future Work

BERT and ChatGPT have both proven effective in classifying personal accounts of LKD on platforms such as Reddit, achieving approximately 80% accuracy, which increases to about 90% when considering acceptable errors, marking a step forward in using web-based data for LKD research. These models could potentially automate the screening of new content for further scrutiny, thereby aiding donor support initiatives, particularly in education and community outreach. Despite the promising results, the complexity of the subject matter complicates the task of making perfect predictions. Our initial attempts to use fine-grained classifications led to suboptimal results, requiring us to use coarse-grained categories. Regarding costs, BERT’s open-source nature and the flexibility to fine-tune make it an appealing choice. In contrast, ChatGPT excels in providing understandable reasoning for its decisions.

A review of errors indicated that ChatGPT generally understood the context well, although there were instances where the reasoning was off the mark, highlighting the importance of clear, prompt instructions. Interestingly, there were instances where the LLMs’ reasoning surpassed ours, especially in delineating the “past” and “present” boundary, thereby suggesting a potential for iterative prompt enhancements informed by LLM reasoning. However, the quest for prompt optimization (or “promptization,” if you will) may present an unending journey, as the allure of “just one more experiment” to elevate performance is always present. Drawing a line on performance as “good enough” is crucial, which may be attained through automated processes, as explored in some recent and exciting studies [63-69]. Future work will leverage these powerful new methodologies to both improve performance on our coarse-grained 3-class schema as well as achieve superior performance on the fine-grained 6-class schema that was unattainable with the present methods.

The performance of both models is significantly constrained by the size of the available data. While thousands of Reddit posts related to LKD are accessible, only a fraction pertains to personal experiences. The performance consistency across different data folds for BERT and across different sample sizes for ChatGPT highlights the need for larger datasets to better gauge each model’s robustness.

A core challenge lies in the task’s inherent demand for a singular label, which often oversimplifies the nuanced narratives in internet posts. Future endeavors could explore more elaborate information extraction techniques, leveraging LLMs such as ChatGPT to answer multiple queries or even construct knowledge graphs per post. Although ensuring uniform and usable output formats remains a hurdle, our work underscores ChatGPT’s proficiency in deriving insightful inferences from the text. Our findings concerning the influence of few-shot learning examples on output bias also suggest the need for deeper investigation into the interplay between example selection and model performance.

With reliable automation methods that can identify when a person is describing a personal experience with LKD, future work will extend the reach to additional media platforms, each of which has its own system for reaching users via advertising. There will certainly be potential biases in accessing educational information about living donations based on the characteristics of audiences most likely to post on each platform. To not exacerbate disparities, one must examine the generalizability of the profiles across multiple platforms and ensure the dissemination of information across platforms that reach diverse audiences and non-English speakers. An examination of access to most audience members, particularly the underserved, is warranted to ensure that all communities are reached equitably.

### Utility of Results

By identifying these unique user classifications, tailored educational interventions for different profiles could be designed. First, for those most actively considering living donation, there could be social media campaigns built and targeted to specific users to invite them to learn more about living donation. These users can be referred to a trusted site, which includes education materials and an opportunity to register to begin donor medical evaluation at a nearby transplant center [41,42]. For individuals discussing their concerns about the costs involved with becoming a living donor, referrals to websites that discuss the ways to apply for grants to cover the out-of-pocket costs and lost wages could be valuable in their decision-making [70].

Second, for donors and families identified to have completed donations, campaigns inviting them to share their experiences on a living donor storytelling website [8,9] might result in more real-life stories being captured from diverse individuals to increase awareness of living donations for the national public. Stories are particularly valuable for educating learners with low health literacy or those for whom English is not their primary language about the possibilities of living donation [71].

Finally, it will be very important to work with experts in marketing and campaign design to plan social media campaigns that are motivating and helpful for patients and their families at different points along their donation journey. Identifying motivated learners from platforms such as Reddit, delivering content to them about living donation, and assessing its impact on learning more or pursuing donation are our next planned steps.

The proposed profiles may incorrectly identify a person’s interest or stage of pursuit of donation, making any educational information sent to them irrelevant. In contrast, users could also be made uncomfortable if the education being provided matches their needs perfectly, indicating that their data are being scrutinized. Users can always disregard nonrelevant content; however, it will be important in the design of new campaigns not to assume with too much certainty that all learners are correctly identified. Respect for users is an ethical tenet that must always be considered in designing the campaigns and communicating how we found that they might be considering living donations as we move forward.

### Conclusions

Much of the previous health care–related research about LLMs has been centered on their reliability in producing quality medical information. In contrast, we endeavor to extract individual-level information from the internet that can be used to inform health care providers. Consequently, there is little comparison that can be made to previous work other than to say that the reliability of the models is subject to the instructions they are given. However, our experimental results do illustrate that when using examples as part of the prompt (few-shot), bias toward the class of the given examples can affect performance. We have also shown that simulating a dialogue between 2 experts is more effective than using stand-alone reasoning.

This study takes a significant step in applying advanced NLP methods to the field of LKD, focusing on automating the detection of personal LKD experiences in online content. Both BERT and ChatGPT proved effective for this task, each with its own advantages and disadvantages. Our new DUCC method outperformed traditional reasoning approaches, emphasizing the importance of further work on improving prompt design. The study also highlights the need for automated prompt creation to reduce the time and effort currently required for manual testing, making NLP applications in the LKD field more efficient and impactful.

## Appendix

Multimedia Appendix 1 Full experimental results.

## Abbreviations

BERT: Bidirectional Encoder Representations from Transformers
DUCC: dialogue until classification consensus
LDKT: living donor kidney transplantation
LKD: living kidney donation
LLM: large language model
NLP: natural language processing
